# Evidence for aggressive mimicry in an adult brood parasitic bird, and generalized defences in its host

**DOI:** 10.1098/rspb.2015.0795

**Published:** 2015-07-07

**Authors:** W. E. Feeney, J. Troscianko, N. E. Langmore, C. N. Spottiswoode

**Affiliations:** 1 Research School of Biology, The Australian National University, Canberra, Australian Capital Territory, Australia; 2 Department of Zoology, University of Cambridge, Cambridge, UK; 3 Centre for Ecology and Conservation, University of Exeter, Penryn, UK; 4 DST-NRF Centre of Excellence at the Percy FitzPatrick Institute, University of Cape Town, Cape Town, South Africa

**Keywords:** brood parasite, coevolution, cuckoo finch, egg rejection, evolution, plumage mimicry

## Abstract

Mimicry of a harmless model (aggressive mimicry) is used by egg, chick and fledgling brood parasites that resemble the host's own eggs, chicks and fledglings. However, aggressive mimicry may also evolve in adult brood parasites, to avoid attack from hosts and/or manipulate their perception of parasitism risk. We tested the hypothesis that female cuckoo finches (*Anomalospiza imberbis*) are aggressive mimics of female *Euplectes* weavers, such as the harmless, abundant and sympatric southern red bishop (*Euplectes orix*). We show that female cuckoo finch plumage colour and pattern more closely resembled those of *Euplectes* weavers (putative models) than *Vidua* finches (closest relatives); that their tawny-flanked prinia (*Prinia subflava*) hosts were equally aggressive towards female cuckoo finches and southern red bishops, and more aggressive to both than to their male counterparts; and that prinias were equally likely to reject an egg after seeing a female cuckoo finch or bishop, and more likely to do so than after seeing a male bishop near their nest. This is, to our knowledge, the first quantitative evidence for aggressive mimicry in an adult bird, and suggests that host–parasite coevolution can select for aggressive mimicry by avian brood parasites, and counter-defences by hosts, at all stages of the reproductive cycle.

## 1. Introduction

Avian brood parasites lay their eggs in the nests of other birds, foisting the cost of parental care onto the host. Hosts often evolve defences against brood parasitism, which in turn selects for counter-offences in brood parasites and further counter-defences in hosts [1]. These relationships generate remarkable and diverse reciprocal adaptations and counter-adaptations, and comprise among the best-characterized examples of coevolution in nature [2,3].

Adaptations to circumvent host defences in brood parasites are often deceptive. Mimicry of a harmless model (aggressive mimicry [4]) is a commonly employed form of deception, and is evident in egg [5,6], chick [7,8] and fledgling [9] brood parasites that resemble the eggs, chicks and fledglings of their hosts. Mimicry at these stages of the nesting cycle increases the likelihood of acceptance of the brood parasite by the foster parents [6,9,10]. However, many hosts are hostile towards adult brood parasites [11] and are more likely to reject a foreign egg after the sight of an adult parasite near their nest [12,13]. This suggests that aggressive mimicry may also be beneficial to adult brood parasites.

**Figure 1.**
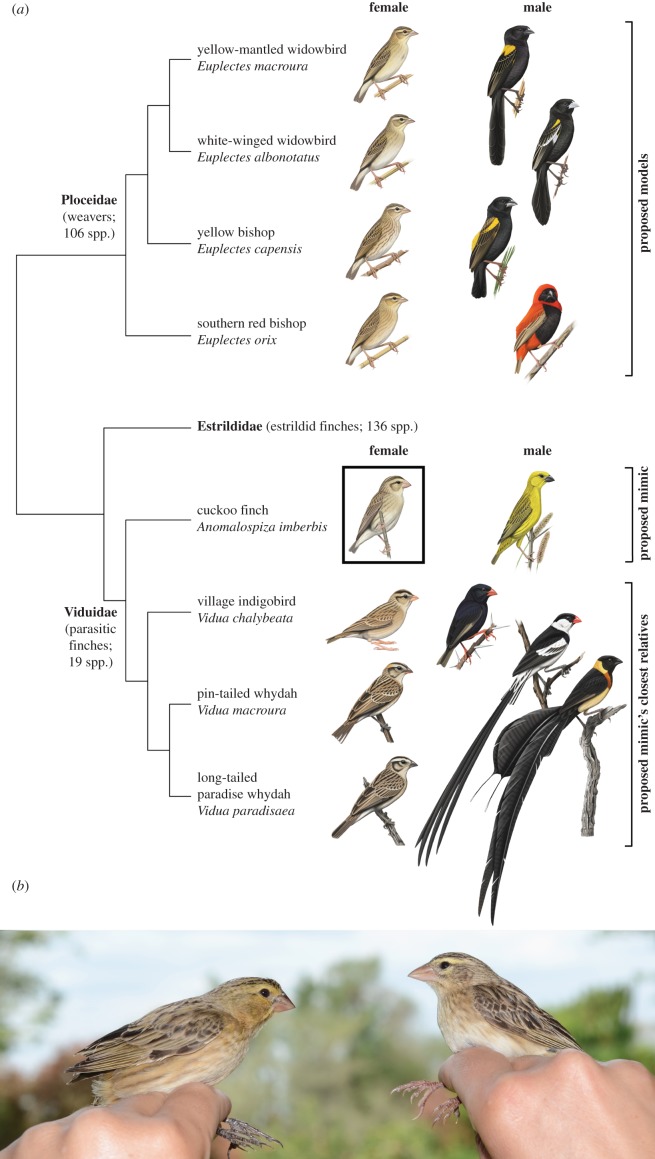
(*a*) Relatedness between *Euplectes* weavers and the parasitic finches used in this study. Phylogenetic data were obtained from [21–23], and illustrations were reproduced with permission from Faansie Peacock [19]. (*b*) Female cuckoo finch (left), and female southern red bishop (right) caught in Choma, Zambia (photograph by C.N.S.).

**Figure 2.**
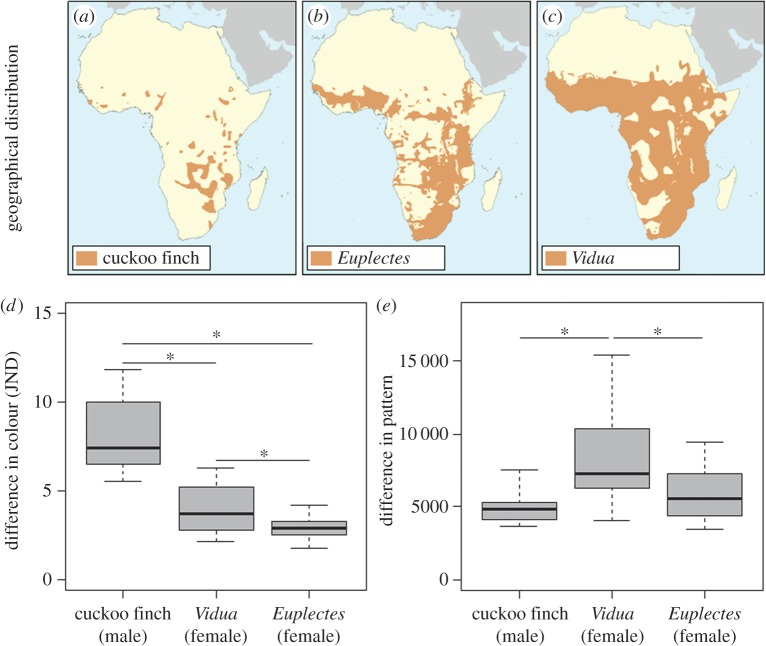
The geographical distribution of the (*a*) cuckoo finch, (*b*) *Euplectes* (*Euplectes albonotatus*, *E. capensis*, *E. macroura* and *E. orix*) weavers and (*c*) *Vidua* (*Vidua chalybeata*, *V. macroura* and *V. paradisaea*) finches that were analysed in this study. Throughout sub-Saharan Africa, 82% of the cuckoo finch's distribution lies within that of the (merged) *Euplectes* weavers’ distribution, and within 96% of the (merged) *Vidua* finches’ distribution. Range map data were kindly provided by BirdLife International and NatureServe [24]. Note that these percentages are underestimates as we only included *Euplectes* and *Vidua* finch species that were present at our study site in southern Zambia, and had a minimum of eight skin samples available for analysis at The Natural History Museum at Tring, UK. Differences in (*d*) colour (JND, just notable difference) and (*e*) pattern between cuckoo finch female plumage and cuckoo finch male (*Anomalospiza*), and sympatric *Vidua* and *Euplectes* species. Asterisks denote significant differences and whiskers show ranges. *p*-values for pairwise comparisons were obtained by varying the reference category in the models. Summary data are presented in the electronic supplementary material, table S1.

Morphological aggressive mimicry has been suggested to occur in adult birds [14–16], but has never been quantitatively investigated (for an example of vocal aggressive mimicry, see [17]). Of the possible candidates, the brood-parasitic cuckoo finch (*Anomalospiza imberbis*) provides a good model to test for it. Female cuckoo finches bear a striking resemblance to abundant, sympatric and harmless female *Euplectes* weavers [15,18–20] (figures 1*a*,*b* and 2*a,b*). In fact, prior to a phylogenetic analysis that placed it sister to the *Vidua* finches (Viduidae) [21], the cuckoo finch was often considered a member of the weaver family (Ploceidae) (reviewed by Lahti & Payne [25]). Aggressive mimicry is only beneficial when the mimic is rare compared with its model [26], and cuckoo finches are rare compared with *Euplectes* weavers: within their respective southern African ranges, the mean reporting rate for cuckoo finches was 1.3% compared with 13.6–32.4% for the *Euplectes* weavers considered in this study [27]. Cuckoo finches (putative mimic), *Euplectes* weavers (putative models) and *Vidua* finches (closest relatives) overlap in their distributions (figure 2*a*–*c*), inhabit a similar range of grasslands/grassy savannahs and are variably social granivores [18,19,27]. This provides an opportunity to compare the plumage of a putative mimic with that of both its closest relatives and its putative models, which all live in the same habitat and occupy comparable ecological niches. These species are also sexually dichromatic (figure 1*a*), allowing for behavioural experiments to test whether hosts can distinguish female cuckoo finches from *Euplectes* weavers compared with their dissimilar-looking male counterparts. Finally, there is established evidence of coevolution between the cuckoo finch and its primary host in southern Zambia, the tawny-flanked prinia (*Prinia subflava*: hereafter ‘prinia’) [6,28–31]; it is therefore plausible to suspect that anti-parasitic nest defence by hosts might have selected for aggressive mimicry in adult female parasites.

The aim of this study was to test the hypothesis that adult female cuckoo finches use aggressive mimicry of *Euplectes* weavers to deceive their hosts. This hypothesis predicts that cuckoo finches should look like *Euplectes* weavers, and that hosts should behave similarly in response to cuckoo finches and to *Euplectes* weavers compared with controls. First, we quantified the resemblance between cuckoo finches and *Euplectes* weavers using museum skins, and sought to distinguish between the several different processes that could generate shared appearance. If the plumage of female cuckoo finches is a product of shared ancestry, it should more closely resemble that of *Vidua* finches (closest relatives) than *Euplectes* weavers (putative models). If it arises from convergent evolution resulting from shared ecological pressures (e.g. crypsis in grassy habitats), then the three taxa should be uniform in appearance. By contrast, if it reflects aggressive mimicry resulting from brood parasite–host coevolution, then we should expect female cuckoo finches to resemble *Euplectes* weavers (putative models) more closely than *Vidua* finches (closest relatives).

Next, using model presentation experiments at prinia nests, we investigated whether host parents distinguished female and male cuckoo finches (i.e. putative mimic and dissimilar-looking male) from female and male southern red bishops (*Euplectes orix*, a common *Euplectes* weaver at our study site, hereafter ‘bishop’; i.e. putative model and dissimilar-looking control). While aggression towards an adult brood parasite is not a brood parasite-specific defence [32], it is commonly deployed [11] and can deter parasitic egg-laying [33,34]. We predicted that if adult female cuckoo finches are aggressive mimics of adult *Euplectes* weavers, and if hosts recognize adult male cuckoo finches as a threat (suspected following [1,35], C. Moya & R. Munkombwe 2013, personal communication) and have not evolved counter-adaptations against aggressive mimicry, then prinias should be more aggressive towards male cuckoo finches than towards female cuckoo finches, female bishops and male bishops in the vicinity of their nests. Alternatively, if hosts recognize male cuckoo finches as a threat and have evolved counter-adaptations against aggressive mimicry, then prinias should be equally aggressive towards male and female cuckoo finches as well as towards female bishops, and more aggressive towards them than towards male bishops in the vicinity of their nests. If adult female cuckoo finches are aggressive mimics of adult *Euplectes* weavers and if hosts do not recognize adult male cuckoo finches as a threat, prinias should be equally aggressive to female cuckoo finches and female bishops. Again, whether this level of aggression is higher than, or similar to that received by male cuckoo finches and bishops will depend on whether or not hosts have successfully evolved generalized counter-defences against potentially brood-parasitic intruders. If prinias do not recognize the female cuckoo finch as a threat/aggressive mimic, then they should show low levels of aggression towards all model types.

Finally, after finding that prinias were equally aggressive towards female cuckoo finches and female bishops, but not towards their male counterparts near their nests (see Results), we conducted coupled model presentation and egg rejection experiments to test whether prinias exhibited brood parasite-specific defences towards both female cuckoo finches and female bishops. Egg rejection is a brood parasite-specific defence [36], and the decision to reject an egg from the nest can be affected by the perceived risk of brood parasitism [12,13]. We presented prinias with a female cuckoo finch, female bishop or male bishop, and then experimentally parasitized the prinia nests with a foreign egg. As a control, we used a male bishop, because it received the weakest response in the previous experiment (see Results). If prinias cannot distinguish between putative mimics and models, then we should expect that prinias would show a similar rate of egg rejection after seeing a female cuckoo finch or a female bishop model near their nest. This rate of egg rejection in response to female cuckoo finches and female bishops may be similar to that shown in response to male bishops if prinias have not evolved counter-adaptations to aggressive mimicry, or may be higher if hosts have evolved generalized counter-defences against aggressive mimicry.

## 2. Material and methods

### (a) Study site and system

Fieldwork was conducted during January–March 2013, within a *ca* 1700 ha area on and around Musumanene and Semahwa Farms (centred on 16°46′ S, 26°54′ E) in the Choma District of southern Zambia. The habitat comprises miombo woodlands, grasslands and agricultural fields, where prinias are abundant and regularly parasitized by cuckoo finches (at least 19% of nests experience parasitism attempts [6]). Prinias suffer high fitness costs as a result of brood parasitism (cuckoo finches remove at least one egg upon parasitism, and cuckoo finch hatchlings usually outcompete all host young [30,31]), which has selected for host defences including aggression towards adult cuckoo finches [31] and high rates of rejection of foreign eggs [6].

### (b) Analyses of museum skin colour and pattern

To investigate morphological aggressive mimicry of female *Euplectes* weavers by female cuckoo finches, we conducted plumage colour and pattern analyses of museum skins from the Natural History Museum (Tring, UK) in 2013. We compared the colour and pattern of female cuckoo finch plumage to those of male cuckoo finches and all available female *Euplectes* weavers and female *Vidua* finches that occur in sympatry with cuckoo finches at our study site in southern Zambia (*n* = 8 per species): white-winged widowbird (*E. albonotatus*), yellow bishop (*E. capensis*), yellow-mantled widowbird (*E. macroura*), southern red bishop (*E. orix*), village indigobird, (*Vidua chalybeata*), pin-tailed whydah (*V. macroura*) and long-tailed paradise whydah (*V. paradisaea*) (figure 1*a*). The purple indigobird (*V. purpurascens*) and broad-tailed paradise whydah (*V. obtusa*) also occur at our study site, but were not included in our analyses as too few skins were available.

We quantified plumage colour, luminance and pattern from standardized photographs of museum skins. Photographs were taken in RAW format with a Nikon D7000 camera and Micro-Nikkor 105 mm macro lens, and a Metz Mecablitz 76 MZ-5 external flashgun was used for all ultraviolet (UV) photographs (electronic supplementary material, figures S1–S10). The camera was modified by removal of its UV and infrared (IR) blocking filter, which was replaced with a quartz sheet to allow quantification of colour throughout the avian-visible spectrum (Advanced Camera Systems, Norfolk, UK); the flashgun was similarly modified by removing its UV blocking filter. All photographs were linearized and normalized against a 40% reflectance Spectralon grey standard (Labsphere, Congleton, UK) included in each photograph. Visible spectrum photographs were taken through a Baader UV–IR blocking filter (Baader Planetarium, Mammendorf, Germany), permitting only visible spectrum light from 420 to 680 nm, and UV photographs were taken with a Baader UV pass filter permitting only UV light from 320 to 380 nm. Cone catch quanta were modelled from digital images following a widely used polynomial transformation technique [37,38], performed by custom-written code in ImageJ [39].

Photographs of the study skins were taken from dorsal, ventral and lateral viewpoints, and nine patches were selected for analysis (back, beak, belly, breast, cheek, eyebrow, head, throat and wing). As the prinia visual system has not been described, we used the visual system of the UV-sensitive blue tit (*Cyanistes caeruleus*) [40] as our model visual system (following [6,28–30]). Owing to the degrading effect of UV light sources on museum specimens, UV photography was only used on a subset of the skins (*n* = 2 females of each *Euplectes* and *Vidua* species, as well as two male and two female cuckoo finches). However, visual inspection of these photographs suggests that these species do not substantially differ in UV reflectance (electronic supplementary material, figures S2–S10). Therefore, museum skin colour analyses were based on trichromatic ‘just notable difference’ (hereafter ‘JND’) [41] calculations according to Weber fractions for short-wave sensitive, medium-wave sensitive and long-wave sensitive cone types [40], using the camera's vRGB (visible pass filter) images. From this, we calculated the single pixel colour combination that had more other pixels in the image within two JNDs of colour difference than any other colour (i.e. to calculate most ‘abundant’ colour in each body region) for analysis. Images of each patch were scaled down to 2000 pixels to accommodate the exhaustive calculation process (see the electronic supplementary material, table S1, for summary data).

Plumage pattern and luminance analyses were performed on double cone responses because pattern is thought to be encoded primarily by achromatic vision [42]. Luminance distribution differences (*L*_diff_) were calculated from comparing absolute differences in counts of the numbers of pixels in each body region (e.g. body region A in the cuckoo finch compared with the corresponding body region A in one of the other specimens) at 32 linear levels of luminance from 0 to 100%:
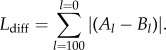
*L*_diff_ values reflect the similarity between two samples’ perceived brightness, allowing for plumage patterns that create a non-normal luminance distribution. Spatial frequency differences were generated using fast Fourier transform bandpass filters at 33 levels, using the standard deviation of pixel intensity values at each spatial scale to represent the ‘energy’ at that spatial scale. Spatial frequency differences (*S*_diff_) were calculated in a similar manner to *L*_diff_, by summing the absolute differences in energy between corresponding body regions at each spatial scale:
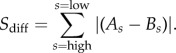


Any differences in pattern energy between the samples at any spatial scale will increase the *S*_diff_ value. Thus, *S*_diff_ describes the degree to which body region patterns match in their size and spacing and the differences in contrast between their patterns.

When comparing two similar patterns, this approach has a number of advantages over previous methodologies that separate out the energy spectra into multiple descriptive statistics [6,28,29,43]. For example, spatial energy spectra can often be complex and multi-modal, so selecting only the peak frequency or peak energy discards much of that potentially important pattern information at other scales, and can erroneously switch between peaks in a multi-modal distribution. Combining pattern similarity into a single measure also makes statistical analysis more straightforward. Similar to our colour JND values, luminance JND values were calculated according to mean luminance pixel values (following [44]).

### (c) Model presentation experiments

We used model presentation experiments to investigate whether prinias could distinguish between female cuckoo finches and *Euplectes* weavers. We presented 15 prinia breeding pairs with models of a female cuckoo finch (putative mimic), male cuckoo finch (dissimilar-looking male), female southern red bishop (putative model) and male southern red bishop (control) at their nests during egg laying or early incubation (i.e. within 3 days of the clutch being completed; similar to [33,45]).

To create our experimental models, specimens (*n* = 2 of each) were caught using mist nets and were sacrificed by chest compression (ANU Animal Ethics Number: A2012/60). The models were then prepared by injecting the specimen with high concentration (more than 80%) of formalin solution. At least 30 min prior to each experiment, a hide was set up within sight (15–30 m) of the nest, and the perch (approx. 1.3 m in height) on which each model was positioned was placed approximately 1.5 m from the nest to allow for host acclimation. The prinia pair was then monitored until they left the area, whereupon a model was placed upon the perch. Each pair experienced all treatments, presentation order was randomized prior to experimentation, model replicate was swapped between trials to test for model effects, and all four models were presented to the same pair on the same day (methods similar to [33,45,46]). Each trial began when a prinia either came within 2 m of the model, or came within 5 m of the model and began alarm calling while facing the model. Each trial continued for 5 min, and a minimum of 60 min was allowed between each presentation to allow for carryover aggression to diminish. Prinia vocalizations were recorded using a Marantz PMD661 solid-state recorder and an Audio-Technica condenser microphone. The time spent mobbing the model (within 0.5 m) by each individual was dictated during each trial for later extraction.

### (d) Coupled model presentation and egg rejection experiments

Following the results of the model presentation experiment, we investigated whether prinia deception by female cuckoo finches affected their subsequent anti-parasitic decision-making, by means of coupled model presentation and egg rejection experiments. We presented 51 prinia breeding pairs with a female cuckoo finch, female bishop or a male bishop model (*n* = 17, 16, 18, respectively): only one model was presented at each prinia nest, and each trial involved a different prinia breeding pair (methods of model presentation experiments outlined above). Following each model presentation, we replaced a prinia egg with an experimental parasitic egg (conspecific prinia egg) to simulate a parasitism attempt [6]. We used conspecific eggs as they are easy to obtain, avoid drawbacks of artificially constructed eggs [47] and have been used successfully in previous egg rejection experiments in this system at this site [6,28,29]. As prinias lay highly polymorphic eggs [6], our experimental parasitic eggs varied in appearance from highly mimetic to non-mimetic relative to the host eggs. All experiments took place during the host laying period (i.e. before clutch completion).

We measured and photographed experimental and host eggs to quantify egg volume, shape and pattern, and measured reflectance spectra of each egg indoors to quantify egg background colour. Egg volume and shape were calculated from the digital images (following [48]) and pattern was quantified following the methods described for measuring plumage pattern (above). The reflectance spectrum of each egg was measured indoors with an Ocean Optics USB2000 spectrophotometer with a PX-2 pulsed xenon light source and an R400–7-UV/VIS reflectance probe (all Ocean Optics), and was standardized using a Spectralon 99% white reflectance standard (Labsphere). Eggs were held at a constant angle (45°) and distance (5 mm) from the probe tip during spectral measurements using an attached plastic sleeve, and five measurements of the background colour (so far as possible avoiding pattern markings) were taken per egg. Egg cone catch quanta were used to calculate colour and luminance JNDs between host and experimental eggs (following [6,28–30]). As we used tetrachromatic blue tit (ultraviolet sensitive) analyses on host and experimental eggs (rather than the trichromatic analyses on museum skins), we have also presented analyses using a violet sensitive visual system (using peafowl vision as a model [49]) in the electronic supplementary material (all conclusions were unchanged).

Following insertion of the experimental egg, experimental clutches were visited as often as possible (the majority were visited daily) to determine whether or not an egg had been rejected. A single missing egg was considered rejected, as predators typically remove an entire clutch; entire clutches that remained intact and under active incubation for 3 days were considered accepted (following [6]).

### (e) Statistical analyses

Audio data from the model presentation experiments were extracted using RavenPro v. 1.3 [50]. Plumage colour, luminance and pattern calculations, and egg colour, luminance, pattern, shape and volume calculations were performed in ImageJ v. 1.47q [39]. Statistical analyses were conducted in R v. 2.13.2 [51], and linear mixed-effects models were conducted using the nlme R package [52].

We used linear mixed-effects models to test whether female cuckoo finch plumage (colour, luminance and pattern) was more similar to that of sympatric female *Euplectes* weaver or *Vidua* finch species. We calculated the mean difference in colour, luminance and pattern across species for analyses, as the data trend for each of these attributes was clear (see the electronic supplementary material, table S1, for summary data). Our full models included treatment (male cuckoo finch, female *Euplectes* weaver or female *Vidua* finch) as a fixed effect and body region as a random effect. Our measure of pattern was log-transformed to satisfy model assumptions. We used linear mixed-effects models to test whether prinia aggression (number of alarm calls and time spent mobbing) varied in response to the four model types (female cuckoo finch, male cuckoo finch, female bishop and male bishop). Our full model included model type, presentation order, model replicate and nest stage (laying versus early incubation) as fixed effects and pair identity as a random effect. The final model included model type as a fixed effect and pair identity as a random effect after removing non-significant factors (at *p* > 0.05). We used a logistic regression to test whether prinia pairs were more likely to reject an experimental egg after seeing a female cuckoo finch, female bishop or a male bishop. Our full model included model type, difference in egg colour, difference in egg luminance, difference in egg pattern, differences in egg shape and differences in egg volume as fixed effects. The final model included model type and difference in egg colour after removing non-significant factors (at *p* > 0.05). All conclusions were unchanged when instead using Akaike information criterion-based model selection. Full and final model outputs are presented in the electronic supplementary material, tables S2–S7.

## 3. Results

### (a) Museum skin analyses

The plumage of the female cuckoo finch more closely resembled that of their putative models, the *Euplectes* weavers, than that of their closest relatives, the *Vidua* finches, supporting a hypothesis of aggressive mimicry rather than shared ancestry or shared ecology. When modelled through a bird's eye, the similarity in plumage luminance between the female cuckoo finch and female *Euplectes* weavers was not different from the similarity in plumage luminance between the female cuckoo finch and male cuckoo finch (linear mixed effects model (LME): 
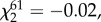
 *p* = 0.78), or between the female cuckoo finch and the female *Vidua* finches (LME: 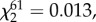 *p* = 0.81). By contrast, the female cuckoo finch and female *Euplectes* weavers were significantly more similar to one another in plumage colour than was the female cuckoo finch to either the female *Vidua* finches (LME: 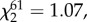 *p* = 0.02; figure 2*d*) or male cuckoo finch (LME: 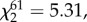 *p* < 0.0001; figure 2*d*). Likewise, while the similarity in plumage pattern between the female cuckoo finch and the female *Euplectes* weavers was not different from the similarity in plumage pattern between the female cuckoo finch and the male cuckoo finch (LME: 
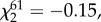
 *p* = 0.32; figure 2*e*), the degree of similarity within each of these pairs was greater than the degree of similarity between the female cuckoo finch and the female *Vidua* finches (LME: 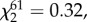 *p* = 0.0027; figure 2*e*). In summary, female cuckoo finches look more similar in colour to female *Euplectes* weavers than either to conspecific males or to females of their closest relatives; more similar in pattern to *Euplectes* weavers than to females of their closest relatives; and plumage luminance did not significantly differ across taxa. Therefore, these data suggest that female cuckoo finch plumage colour and pattern are not an artefact of common ancestry or convergent evolution resulting from shared ecological pressures, but are consistent with a hypothesis of brood parasite–host coevolution.

Avian visual modelling predicted that prinias would not be able to distinguish the colour of female cuckoo finch plumage from that of female *Euplectes* weavers in good lighting conditions (mean JND colour = 2.86 ± 0.24), but would be able to distinguish it from that of female *Vidua* finches (mean JND colour = 3.92 ± 0.27) and male cuckoo finches (mean JND colour = 8.17 ± 1.46) (figure 2*d*). Prinias should also be unable to distinguish the luminance of female cuckoo plumage from that of female *Euplectes* weavers (mean JND luminance = 0.78 ± 0.08), *Vidua* finches (mean JND luminance = 0.77 ± 0.05) and male cuckoo finches (mean JND luminance = 0.76 ± 0.10).

### (b) Model presentation experiments

**Figure 3.**
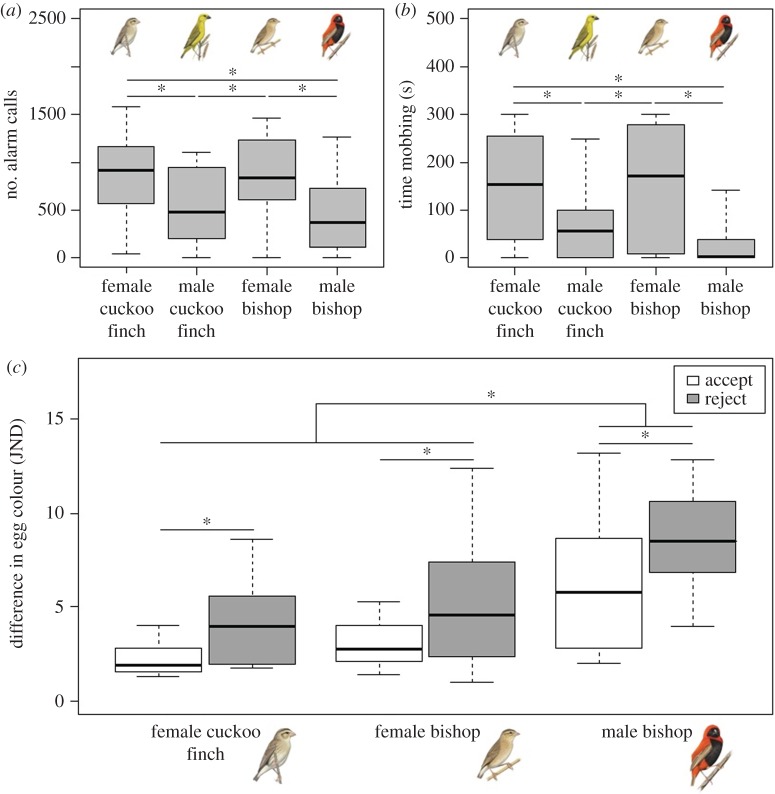
(*a*) Mean number of alarm calls made to each model type: female cuckoo finch, male cuckoo finch, female southern red bishop and male southern red bishop. (*b*) Time spent mobbing (within 50 cm) each model by at least one prinia during the 300 s trial. (*c*) Mean colour difference (measured in JNDs) of accepted and rejected experimental eggs following presentation of a female cuckoo finch, female bishop or male bishop. *p*-values for pairwise comparisons were obtained by varying the reference category in the models. Asterisks denote significant differences and whiskers show ranges.

In accordance with the visual modelling results, prinias did not distinguish between an adult female cuckoo finch and female bishop near their nest. There was no difference in the number of alarm calls they made towards a female cuckoo finch compared with a female bishop (
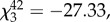
 *p* = 0.80; figure 3*a*), and nor was there a difference in the amount of time prinias spent mobbing the two models (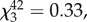 *p* = 0.99; figure 3*b*). However, prinias were more aggressive towards the females of both species than they were to a male cuckoo finch and a male bishop: prinias spent significantly more time mobbing, and made more alarm calls towards a female cuckoo finch than either a male cuckoo finch (
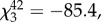
 *p* = 0.0008; 
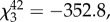
 *p* = 0.0017, respectively) or a male bishop (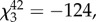 *p* < 0.0001; 
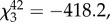
 *p* = 0.0003, respectively) (figure 3*a*,*b*). Although not quantified, in the majority of trials we also observed prinias physically attacking the female cuckoo finch and female bishop models; this degree of physical aggression was never observed during either of the male model trials. Taken together, these results suggest that hosts have evolved generalized counter-defences against potentially parasitic intruders.

### (c) Coupled model presentation and egg rejection experiments

Prinias again demonstrated no evidence of distinguishing an adult female cuckoo finch from an adult female bishop when presented with a foreign egg as well as a model adult intruder and exhibited a brood parasite-specific defence towards both the female bishop and cuckoo finch. Prinia pairs were equally likely to reject a foreign egg after seeing a female cuckoo finch or a female bishop model near their nest (logistic regression: estimate ± s.e. = −0.06 ± 0.75, *Z* = −0.085, *p* = 0.93; 58.2% and 62.5% of prinia pairs rejected an egg after presentation of a female cuckoo finch (*n* = 17) and female bishop (*n* = 16) model, respectively; figure 3*c*). They were significantly more likely to reject an egg after seeing either of these two models than after seeing a male bishop (logistic regression: estimate ± s.e. = −2.05 ± 0.95, *Z* = −2.168, *p* = 0.03; 38.9% of prinia pairs rejected an egg after presentation of a male bishop (*n* = 18) model; figure 3*c*), suggesting that cuckoo finches do not currently benefit from resembling female bishops. Replicating previous studies [6,30], rejected eggs were significantly more different in colour from the host's own eggs than were accepted eggs (logistic regression: estimate ± s.e. = 0.29 ± 0.12, *Z* = 2.365, *p* = 0.018; figure 3*c*). Prinias correctly rejected the introduced egg in all but two cases (12.5%, *n* = 16), in which they mistakenly rejected their own egg, both of which followed a female bishop presentation.

## 4. Discussion

Our data suggest that female cuckoo finches are aggressive mimics of female *Euplectes* weavers and that their prinia hosts have responded to successful deception with generalized defences. First, analyses of museum skins showed that the colour and pattern of female cuckoo finch plumage more closely resemble those of *Euplectes* weavers than those of the cuckoo finch's sister taxon, the *Vidua* finches. This suggests that their resemblance is not an artefact of common ancestry or convergent evolution resulting from shared ecological pressures, but a result of brood parasite–host coevolution (figure 2*d*,*e*). Second, analysis of perceived differences in plumage colour suggested that prinias should not be able to distinguish female cuckoo finches from female *Euplectes* weavers, but should be able to distinguish them from female *Vidua* finches (figure 2*d*). Third, model presentation experiments showed that prinias did not distinguish female cuckoo finches from female bishops and were more aggressive towards both than towards their dissimilar-looking male counterparts (figure 3*a*,*b*). Finally, our coupled model presentation and egg rejection experiments again showed that prinias did not distinguish female cuckoo finches from female bishops: they increased their rate of egg rejection equally after seeing either species near their nest, compared with their rate of egg rejection after seeing a male bishop (figure 3*c*).

Aggressive mimicry is a commonly employed form of deception in avian brood parasites, and may be more common in adults than is currently appreciated. Brood parasites that usurp the entire parental effort of their host rely heavily on deception for host manipulation [3]: detection of an adult parasite can result in increased rates of nest vigilance [53,54], mobbing [34], egg rejection [12,13] and chick rejection [55] by the host. Brood parasites can decrease the likelihood of being detected by behaving cryptically [56] or looking dangerous (Batesian mimicry; [57]), and possibly through looking cryptic [15] or looking harmless (this study). Mimicry of predatory raptors appears to be relatively common among old world cuckoos [58], and correspondingly, there are additional suspected cases of aggressive mimicry in adult brood parasites: brown-backed honeybirds (previously called sharp-billed honeyguides) (*Prodotiscus regulus*) are ‘difficult to distinguish in size, colour, form and behaviour from small grey flycatchers living in the same habitat’ [15], and drongo cuckoos (*Surniculus lugubris*) demonstrate a ‘close visual similarity’ to drongos (*Dicrurus* spp.), ‘in terms of the black adult plumage, white-spotted juvenile plumage and body proportions' [16]. These suspected cases await formal investigation, but taken together with the results of this study, they suggest that host defences can drive aggressive mimicry in avian brood parasites at all stages of their life cycle.

There are several possible explanations for why we did not identify a benefit of mimicry in this study. First, we should expect hosts to vary their response to both model and mimic according to their perceived risk of brood parasitism, as the success of aggressive mimicry is frequency-dependent [26] and as our results suggest that prinias are unable to discriminate between model and mimic. When the risk of parasitism is high, prinias should respond defensively towards female cuckoo finches and female bishops near their nest, as this is where brood parasites pose the greatest threat. When the risk of parasitism is low, prinias should respond towards neither, as defences carry costs. For example, mistaken identification of female bishops resulted in hosts rejecting their own egg in two of 16 trials (12.5%). At our study site, the rate of parasitism is consistently high (approx. 19% parasitism per year [6]), which may explain why prinias responded defensively towards both female cuckoo finches and female bishops. Given the costs of defences, we would not expect prinias to reject eggs as readily after seeing females of either species near their nest at sites where the risk of parasitism is lower. In such settings, mimicry should confer greater benefits to cuckoo finches.

Cuckoo finches may also benefit from aggressive mimicry in other circumstances. First, they may evade recognition and mobbing by naive hosts. For example, parasite-naive superb fairy-wrens *Malurus cyaneus* do not respond to cuckoos [45,46], and young individuals are parasitized more often than their older counterparts, possibly because they are less able to defend themselves against brood parasites, or have not learnt to recognize them [46,59]. The mimetic plumage of female cuckoo finches may similarly facilitate parasitism of cuckoo finch-naive prinias, but an individually marked population of prinias of known age and experience would be required to test this possibility. Second, aggressive mimicry may confer benefits at distances further from the host nest than we tested in our experiments (1.5 m). While other brood parasite species monitor host behaviour from concealed perches in nearby trees [60,61], cuckoo finches must seek host nests in open grasslands and savannahs [27,31]. In such exposed circumstances, resembling an abundant and harmless model may allow female cuckoo finches to remain unrecognized when monitoring host nests from vantage points at medium range. We intended to test this latter possibility, but the lack of an aggressive response towards the male cuckoo finch treatment in our model presentation experiment removed our treatment for comparison and voided this prospect.

In summary, our data support the hypothesis that female cuckoo finches have evolved a plumage that mimics that of common and harmless *Euplectes* weavers. Our results also suggest that, at least at this particular site, prinias have overcome this deception with generalized defences towards female cuckoo finches and similar-looking female bishops close to their nest. Taken together with evidence of aggressive mimicry in brood-parasitic eggs [5,6], chicks [7,8] and fledglings [9], these results suggest that coevolutionary interactions between avian brood parasites and their hosts can select for aggressive mimicry in brood parasites at all stages of their reproductive cycle, and in turn select for a succession of counter-adaptations in hosts.

## Supplementary Material

Table S1-7, Fig. S1-10

## Data accessibility

Data available from the Dryad Digital Repository: http://dx.doi.org/10.5061/dryad.6n8tb.
