# Supplementary material for: Evidence for aggressive mimicry in an adult brood parasitic bird, and generalized defences in its host
Source: Proc Biol Sci. 2015 Jul 7;282(1810):20150795. doi: 10.1098/rspb.2015.0795 (PMC4590487; doi:10.1098/rspb.2015.0795)
Supplement: Table S1-7, Fig. S1-10 [file rspb20150795supp1.docx]

**Electronic Supplementary Material**

*Egg Rejection Experiments* (*VS Visual System*)

Prinia pairs were equally likely to reject a foreign egg after seeing a female cuckoo finch or a female bishop model near their nest (logistic regression: estimate = -0.11, Z = -0.155, P = 0.87), and significantly more likely to reject an egg after seeing those two models than after seeing a male bishop (logistic regression: estimate = -1.73, Z = -1.93, P = 0.05). While not significant, there was a trend for rejected eggs to be more different in colour from the host's eggs than were accepted eggs (logistic regression: estimate = 0.21, Z = 1.847, P = 0.06).

**Table S1.** Summary of difference in luminance, pattern and colour as compared to female cuckoo finch plumage, sorted by species and body region (N = 8 for each body region).

| Species | Body region | Luminance | | | Pattern | | | Colour | | |
| --- | --- | --- | --- | --- | --- | --- | --- | --- | --- | --- |
|  |  | Mean | ± | SE | Mean | ± | SE | Mean | ± | SE |
| *A. imberbis* | back | 0.48 | ± | 0.07 | 1877.89 | ± | 301.51 | 2.65 | ± | 0.5 |
| (male) | beak | 0.99 | ± | 0.18 | 6228.09 | ± | 2667.73 | 4.81 | ± | 0.87 |
|  | belly | 0.56 | ± | 0.08 | 4787.53 | ± | 955.75 | 12.51 | ± | 1.83 |
|  | breast | 0.62 | ± | 0.08 | 3610.73 | ± | 390.66 | 10.67 | ± | 1.84 |
|  | cheek | 0.93 | ± | 0.12 | 3679.44 | ± | 831.13 | 10.28 | ± | 1.03 |
|  | chin | 0.69 | ± | 0.09 | 10467.31 | ± | 1000.31 | 14.54 | ± | 0.91 |
|  | eyebrow | 1.18 | ± | 0.17 | 6529.52 | ± | 1134.92 | 9.77 | ± | 2.12 |
|  | head | 1.03 | ± | 0.09 | 4374.39 | ± | 536.57 | 6.09 | ± | 1.26 |
|  | wing | 0.34 | ± | 0.06 | 2918.27 | ± | 483.74 | 2.25 | ± | 0.47 |
| *E. albonotatus* | back | 0.39 | ± | 0.06 | 2741.66 | ± | 456.95 | 2.01 | ± | 0.27 |
|  | beak | 1.38 | ± | 0.13 | 8218.74 | ± | 2195.56 | 3.72 | ± | 0.75 |
|  | belly | 1.40 | ± | 0.14 | 13384.63 | ± | 2117.15 | 5.03 | ± | 0.72 |
|  | breast | 0.95 | ± | 0.12 | 9395.34 | ± | 1297.23 | 2.53 | ± | 0.85 |
|  | cheek | 1.00 | ± | 0.20 | 9605.11 | ± | 1495.11 | 3.26 | ± | 0.61 |
|  | chin | 1.44 | ± | 0.13 | 6531.87 | ± | 1423.15 | 2.36 | ± | 0.45 |
|  | eyebrow | 0.98 | ± | 0.15 | 13911.85 | ± | 2175.95 | 2.60 | ± | 0.67 |
|  | head | 0.45 | ± | 0.09 | 2694.17 | ± | 651.55 | 2.16 | ± | 0.50 |
|  | wing | 0.37 | ± | 0.06 | 3410.89 | ± | 760.57 | 4.20 | ± | 0.42 |
| *E. capensis* | back | 0.42 | ± | 0.07 | 2233.47 | ± | 333.42 | 1.66 | ± | 0.28 |
|  | beak | 1.13 | ± | 0.18 | 4152.76 | ± | 573.03 | 4.57 | ± | 0.74 |
|  | belly | 0.73 | ± | 0.14 | 8097.73 | ± | 1948.51 | 3.75 | ± | 0.57 |
|  | breast | 0.68 | ± | 0.10 | 6636.35 | ± | 534.26 | 2.59 | ± | 0.80 |
|  | cheek | 0.93 | ± | 0.20 | 5007.29 | ± | 969.39 | 3.06 | ± | 0.48 |
|  | chin | 0.69 | ± | 0.15 | 6652.72 | ± | 1036.56 | 2.22 | ± | 0.51 |
|  | eyebrow | 1.07 | ± | 0.17 | 7402.84 | ± | 1880.12 | 2.78 | ± | 0.58 |
|  | head | 0.58 | ± | 0.07 | 2896.91 | ± | 497.64 | 1.90 | ± | 0.43 |
|  | wing | 0.35 | ± | 0.05 | 3600.15 | ± | 632.62 | 2.59 | ± | 0.55 |
| *E. macroura* | back | 0.37 | ± | 0.06 | 1339.34 | ± | 125.15 | 3.30 | ± | 0.21 |
|  | beak | 0.83 | ± | 0.13 | 7221.51 | ± | 2272.86 | 4.38 | ± | 0.37 |
|  | belly | 0.71 | ± | 0.10 | 8451.02 | ± | 961.09 | 2.42 | ± | 0.55 |
|  | breast | 0.53 | ± | 0.09 | 4816.03 | ± | 1043.71 | 2.91 | ± | 0.39 |
|  | cheek | 0.80 | ± | 0.05 | 6783.88 | ± | 1987.95 | 3.24 | ± | 0.59 |
|  | chin | 0.56 | ± | 0.11 | 6760.83 | ± | 1649.79 | 3.06 | ± | 0.61 |
|  | eyebrow | 0.90 | ± | 0.08 | 7437.77 | ± | 403.88 | 2.32 | ± | 0.83 |
|  | head | 0.45 | ± | 0.09 | 1625.48 | ± | 299.94 | 3.43 | ± | 0.56 |
|  | wing | 0.32 | ± | 0.06 | 3173.49 | ± | 677.13 | 3.52 | ± | 0.28 |
| *E. orix* | back | 0.66 | ± | 0.10 | 2838.28 | ± | 567.53 | 1.15 | ± | 0.12 |
|  | beak | 0.94 | ± | 0.15 | 5201.21 | ± | 1079.00 | 3.08 | ± | 0.71 |
|  | belly | 1.31 | ± | 0.15 | 9210.60 | ± | 1655.19 | 4.21 | ± | 0.62 |
|  | breast | 0.79 | ± | 0.19 | 4424.58 | ± | 961.42 | 2.41 | ± | 0.68 |
|  | cheek | 0.75 | ± | 0.14 | 5044.06 | ± | 910.13 | 2.54 | ± | 0.54 |
|  | chin | 0.99 | ± | 0.19 | 5627.12 | ± | 1770.62 | 2.36 | ± | 0.72 |
|  | eyebrow | 0.92 | ± | 0.18 | 4965.25 | ± | 1341.25 | 2.95 | ± | 0.52 |
|  | head | 0.70 | ± | 0.10 | 3480.74 | ± | 559.04 | 1.60 | ± | 0.38 |
|  | wing | 0.62 | ± | 0.09 | 6665.45 | ± | 902.76 | 1.16 | ± | 0.29 |
| *V. chalybeata* | back | 0.73 | ± | 0.11 | 2492.83 | ± | 416.33 | 1.61 | ± | 0.24 |
|  | beak | 0.64 | ± | 0.08 | 5277.83 | ± | 1126.40 | 3.98 | ± | 0.50 |
|  | belly | 0.70 | ± | 0.13 | 11949.46 | ± | 1691.56 | 3.67 | ± | 0.75 |
|  | breast | 0.60 | ± | 0.15 | 5341.31 | ± | 1116.22 | 2.54 | ± | 0.54 |
|  | cheek | 0.70 | ± | 0.15 | 4758.85 | ± | 680.90 | 3.64 | ± | 0.44 |
|  | chin | 1.11 | ± | 0.18 | 5449.35 | ± | 705.01 | 1.94 | ± | 0.35 |
|  | eyebrow | 0.80 | ± | 0.13 | 10734.78 | ± | 1718.65 | 2.26 | ± | 0.67 |
|  | head | 0.81 | ± | 0.08 | 12848.05 | ± | 2646.61 | 2.10 | ± | 0.36 |
|  | wing | 0.44 | ± | 0.10 | 4819.18 | ± | 977.76 | 1.88 | ± | 0.34 |
| *V. macroura* | back | 0.27 | ± | 0.06 | 2578.53 | ± | 313.71 | 5.81 | ± | 0.90 |
|  | beak | 1.03 | ± | 0.12 | 4233.20 | ± | 420.33 | 4.58 | ± | 0.77 |
|  | belly | 0.77 | ± | 0.13 | 10592.96 | ± | 2029.75 | 4.63 | ± | 0.67 |
|  | breast | 0.58 | ± | 0.08 | 6371.52 | ± | 1376.50 | 3.47 | ± | 0.72 |
|  | cheek | 0.77 | ± | 0.13 | 5345.74 | ± | 946.30 | 7.88 | ± | 0.69 |
|  | chin | 0.97 | ± | 0.12 | 7970.38 | ± | 944.31 | 5.05 | ± | 0.76 |
|  | eyebrow | 0.63 | ± | 0.11 | 7268.14 | ± | 1452.13 | 4.07 | ± | 0.66 |
|  | head | 0.50 | ± | 0.09 | 8069.21 | ± | 959.87 | 4.48 | ± | 0.62 |
|  | wing | 0.47 | ± | 0.09 | 4462.00 | ± | 774.56 | 4.71 | ± | 0.59 |
| *V. paradisaea* | back | 0.73 | ± | 0.05 | 6190.29 | ± | 1272.60 | 2.79 | ± | 0.32 |
|  | beak | 1.04 | ± | 0.17 | 4911.97 | ± | 787.43 | 4.68 | ± | 0.92 |
|  | belly | 1.30 | ± | 0.18 | 10706.05 | ± | 1911.45 | 5.00 | ± | 0.82 |
|  | breast | 1.04 | ± | 0.18 | 7360.85 | ± | 1648.80 | 4.57 | ± | 1.01 |
|  | cheek | 0.97 | ± | 0.09 | 14700.52 | ± | 2339.03 | 5.87 | ± | 0.38 |
|  | chin | 0.86 | ± | 0.06 | 8413.09 | ± | 1415.24 | 4.54 | ± | 0.81 |
|  | eyebrow | 0.93 | ± | 0.06 | 19949.59 | ± | 3039.00 | 3.99 | ± | 0.73 |
|  | head | 0.79 | ± | 0.07 | 21013.66 | ± | 2228.35 | 3.04 | ± | 0.42 |
|  | wing | 0.53 | ± | 0.09 | 6474.61 | ± | 769.94 | 3.24 | ± | 0.74 |

**Table S2.** Full model for model presentation experiment (time mobbing). Bold text indicates significant differences.

|  | χ^2^ Value | Std. Error | DF | t-value | P |
| --- | --- | --- | --- | --- | --- |
| (Intercept) | 157.63 | 56.90 | 40 | 2.77 | < 0.01 |
| Male cuckoo finch model | -84.42 | 24.30 | 40 | -3.47 | **< 0.01** |
| Female southern red bishop model | 1.81 | 24.33 | 40 | 0.07 | 0.94 |
| Male southern red bishop model | -122.25 | 24.63 | 40 | -4.96 | **< 0.01** |
| Order of model presentation (1–4) | -4.68 | 7.70 | 40 | -0.61 | 0.55 |
| Model replicate identity (1 or 2) | 8.73 | 25.59 | 40 | 0.34 | 0.73 |
| Nest stage | -22.73 | 44.32 | 13 | -0.51 | 0.62 |

**Table S3.** Final model for model presentation experiment (time mobbing). Bold text indicates significant differences.

|  | χ^2^ Value | Std. Error | DF | t-value | P |
| --- | --- | --- | --- | --- | --- |
| (Intercept) | 151.27 | 25.53 | 42 | 5.93 | < 0.01 |
| Male cuckoo finch model | -85.4 | 23.70 | 42 | -3.60 | **< 0.01** |
| Female southern red bishop model | 0.33 | 23.70 | 42 | 0.01 | 0.99 |
| Male southern red bishop model | -124 | 23.70 | 42 | -5.23 | **< 0.01** |

**Table S4.** Full model for model presentation experiment (alarm calls). Bold text indicates significant differences.

|  | χ^2^ Value | Std. Error | DF | t-value | P |
| --- | --- | --- | --- | --- | --- |
| (Intercept) | 1050.37 | 245.03 | 40 | 4.29 | < 0.01 |
| Male cuckoo finch model | -334.56 | 105.22 | 40 | -3.18 | **< 0.01** |
| Female southern red bishop model | -24.48 | 105.35 | 40 | -0.23 | 0.82 |
| Male southern red bishop model | -419.28 | 106.63 | 40 | -3.93 | **< 0.01** |
| Order of model presentation (1–4) | -53.63 | 33.32 | 40 | -1.61 | 0.12 |
| Model replicate identity (1 or 2) | -5.42 | 110.49 | 40 | -0.05 | 0.96 |
| Nest stage | -109.32 | 189.16 | 13 | -0.58 | 0.57 |

**Table S5.** Final model for model presentation experiment (alarm calls). Bold text indicates significant differences.

|  | χ^2^ Value | Std. Error | DF | t-value | P |
| --- | --- | --- | --- | --- | --- |
| (Intercept) | 869.27 | 110.17 | 42 | 7.89 | < 0.01 |
| Male cuckoo finch model | -352.80 | 105.37 | 42 | -3.35 | **< 0.01** |
| Female southern red bishop model | -27.33 | 105.37 | 42 | -0.26 | 0.80 |
| Male southern red bishop model | -418.20 | 105.37 | 42 | -3.97 | **< 0.01** |

**Table S6.** Full model for coupled model presentation and egg rejection experiments. Bold text indicates significant differences.

|  | Estimate | Std. Error | z value | P |
| --- | --- | --- | --- | --- |
| (Intercept) | -1.15 | 1.07 | -1.08 | 0.28 |
| Female southern red bishop model | -0.65 | 0.89 | -0.73 | 0.47 |
| Male southern red bishop model | -2.64 | 1.12 | -2.36 | **0.02** |
| JND (blue tit) | 0.34 | 0.16 | 2.12 | **0.03** |
| Egg length | -0.97 | 1.01 | -0.96 | 0.34 |
| Egg width (maximum width) | -4.46E | 3.00 | -1.49 | 0.14 |
| Egg volume | 0.02 | 0.02 | 1.56 | 0.12 |
| Egg ellipse deviation | < 0.01 | < 0.01 | 1.27 | 0.21 |
| Egg luminance | 0.80 | 0.61 | 1.31 | 0.19 |
| Egg spatial frequency | < -0.01 | < 0.01 | -0.48 | 0.63 |

**Table S7.** Final model for coupled model presentation and egg rejection experiments. Bold text indicates significant differences.

|  | Estimate | Std. Error | z value | P |
| --- | --- | --- | --- | --- |
| (Intercept) | -0.59 | 0.64 | -0.93 | 0.35 |
| Female southern red bishop model | -0.06 | 0.75 | -0.09 | 0.93 |
| Male southern red bishop model | -2.05 | 0.95 | -2.17 | **0.03** |
| JND (blue tit) | 0.29 | 0.12 | 2.37 | **0.02** |


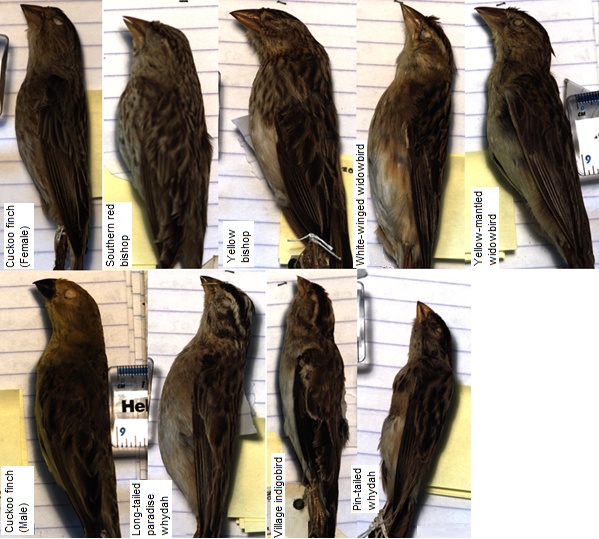


**Figure S1.** Standardized and normalized photographs of species used for plumage colour, pattern and luminance analysis. With exception of the male cuckoo finch, all photographs are of the females of the respective species.


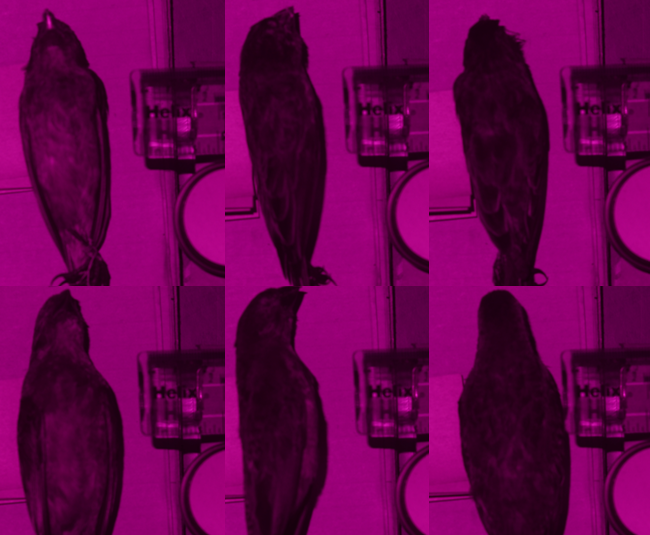


**Figure S2.** UV photograph of two male cuckoo finches


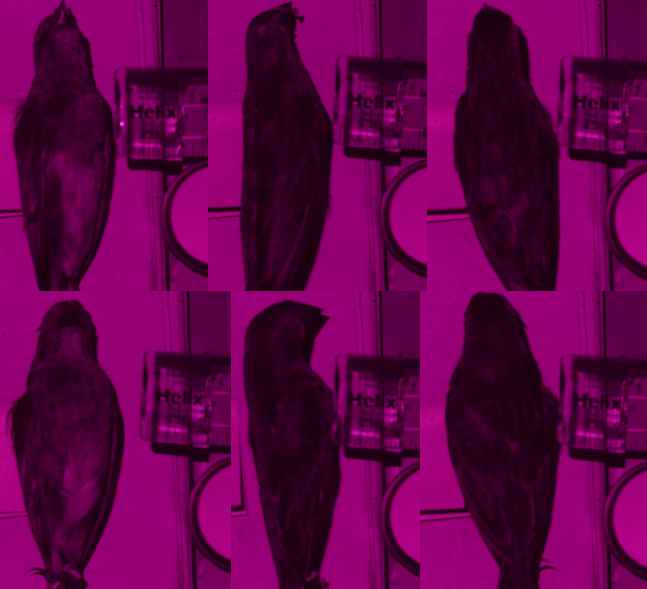


**Figure S3.** UV photograph of two female cuckoo finches


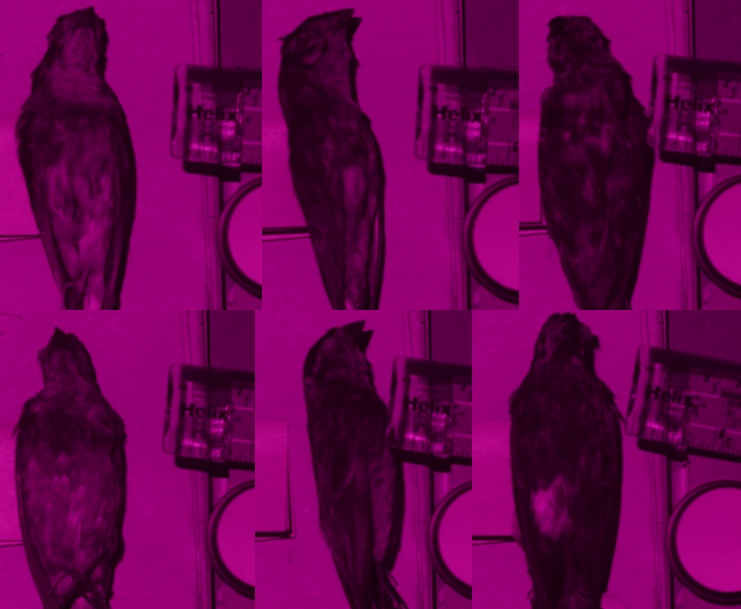


**Figure S4.** UV photographs of female two long-tailed paradise whydahs. The highlighted pale patch is a result of museum stuffing.


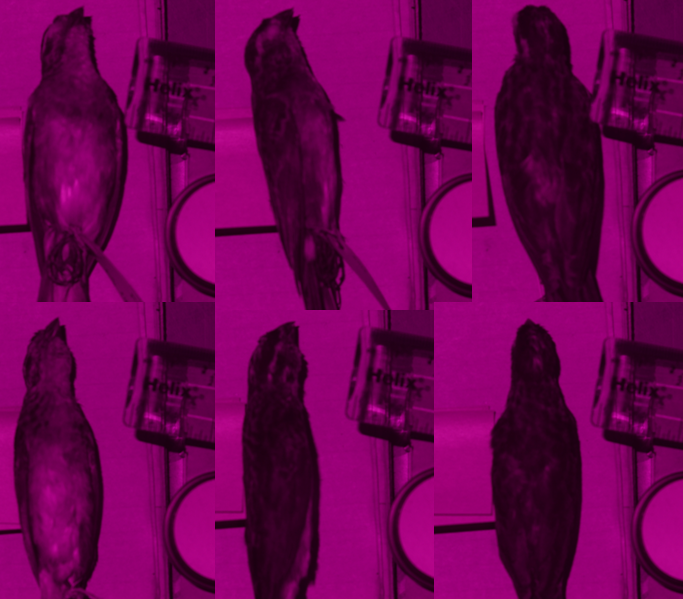


**Figure S5.** UV photographs of female two pin-tailed whydahs.


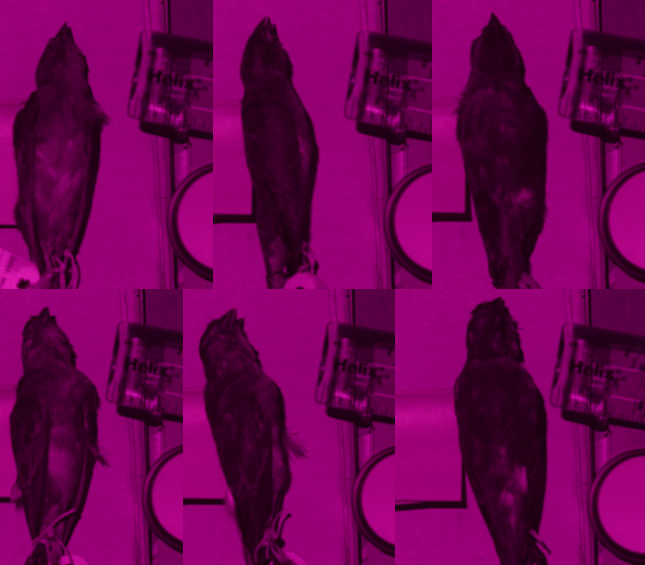


**Figure S6.** UV photographs of two female village indigobirds.


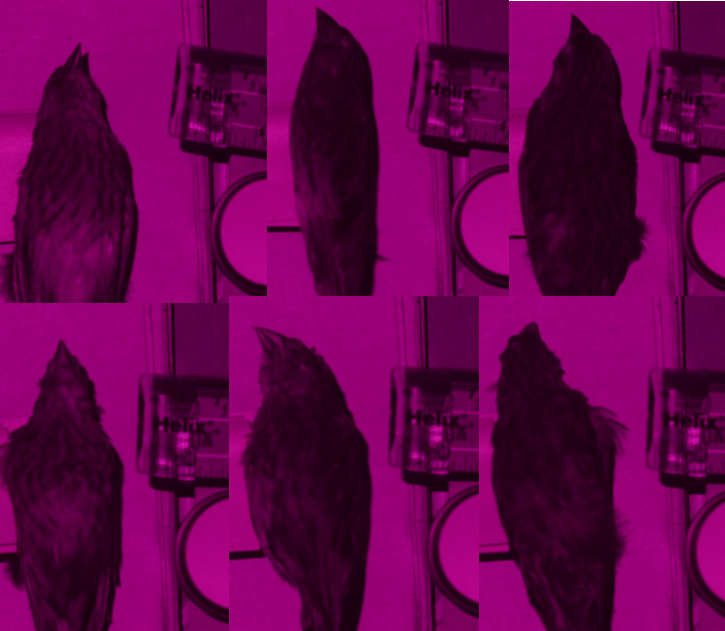


**Figure S7.** UV photographs of two female southern red bishops.


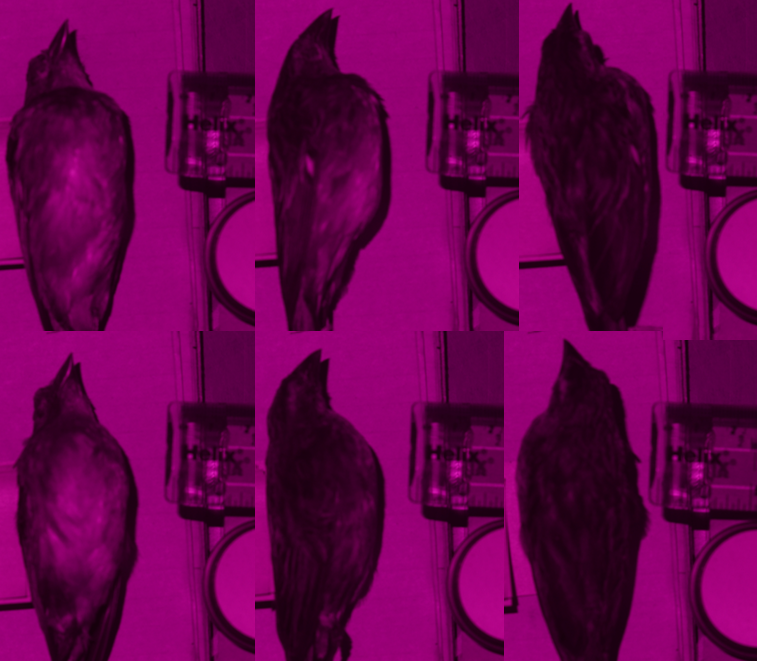


**Figure S8.** UV photographs of two female white-winged widowbirds.


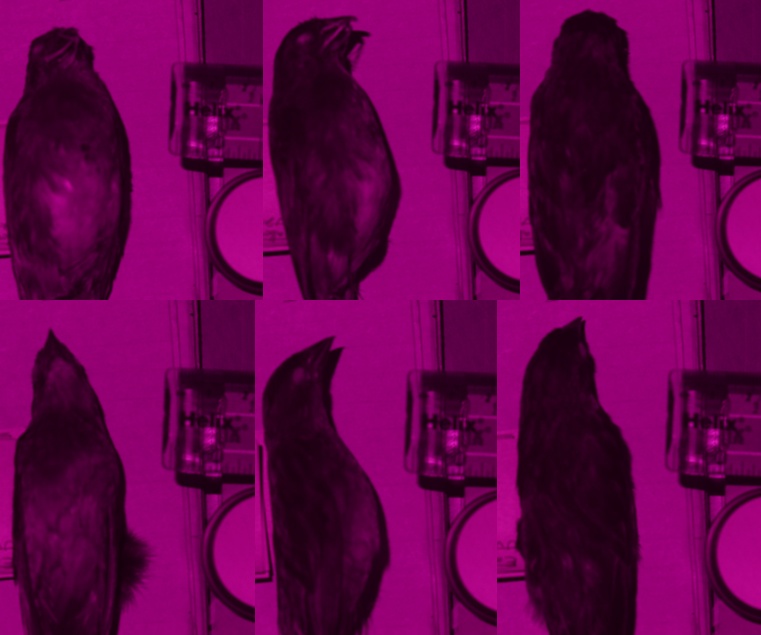


**Figure S9.** UV photographs of two female yellow-mantled widowbirds.


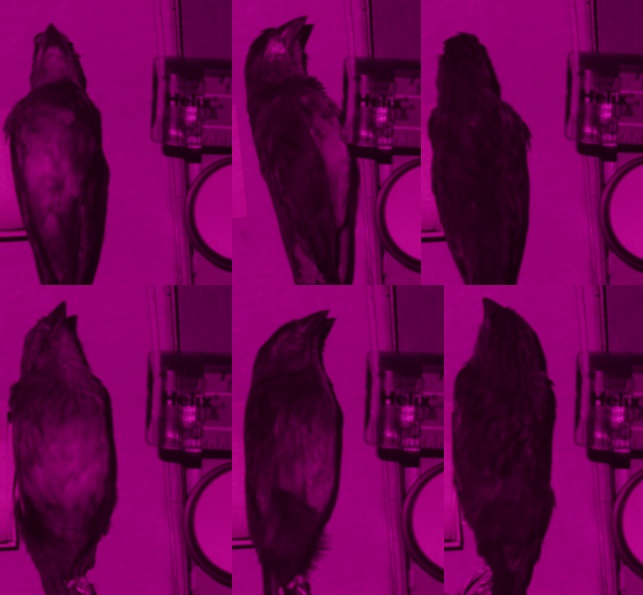


**Figure S10.** UV photographs of two female yellow bishops. The highlighted patch in the top centre photograph is a result of museum stuffing.
